# Cellular and Molecular Mechanisms of Chronic Kidney Disease with Diabetes Mellitus and Cardiovascular Diseases as Its Comorbidities

**DOI:** 10.3389/fimmu.2015.00340

**Published:** 2015-07-08

**Authors:** Prathibha Reddy Gajjala, Maryam Sanati, Joachim Jankowski

**Affiliations:** ^1^Institute for Molecular Cardiovascular Research, Universitätsklinikum RWTH Aachen, Aachen, Germany

**Keywords:** cardiovascular diseases, diabetes mellitus, inflammation, fibrosis, chronic kidney diseases

## Abstract

Chronic kidney disease (CKD), diabetes mellitus (DM), and cardiovascular diseases (CVD) are complex disorders of partly unknown genesis and mostly known progression factors. CVD and DM are the risk factors of CKD and are strongly intertwined since DM can lead to both CKD and/or CVD, and CVD can lead to kidney disease. In recent years, our knowledge of CKD, DM, and CVD has been expanded and several important experimental, clinical, and epidemiological associations have been reported. The tight cellular and molecular interactions between the renal, diabetic, and cardiovascular systems in acute or chronic disease settings are becoming increasingly evident. However, the (patho-) physiological basis of the interactions of CKD, DM, and CVD with involvement of multiple endogenous and environmental factors is highly complex and our knowledge is still at its infancy. Not only single pathways and mediators of progression of these diseases have to be considered in these processes but also the mutual interactions of these factors are essential. The recent advances in proteomics and integrative analysis technologies have allowed rapid progress in analyzing complex disorders and clearly show the opportunity for new efficient and specific therapies. More than a dozen pathways have been identified so far, including hyperactivity of the renin–angiotensin (RAS)–aldosterone system, osmotic sodium retention, endothelial dysfunction, dyslipidemia, RAS/RAF/extracellular-signal-regulated kinase pathway, modification of the purinergic system, phosphatidylinositol 3-kinase (PI 3-kinase)-dependent signaling pathways, and inflammation, all leading to histomorphological alterations of the kidney and vessels of diabetic and non-diabetic patients. Since a better understanding of the common cellular and molecular mechanisms of these diseases may be a key to successful identification of new therapeutic targets, we review in this paper the current literature about cellular and molecular mechanisms of CKD.

## Introduction

A healthy person could be defined as “a man with highly/tightly regulated and coordinated complex biological networks.” The crosstalk between the organs and the systems via molecular, cellular, paracrine, endocrine, and neuronal factors are essential in regulating these networks (1). However, this tight–knit relationship is deregulated at some point when your body goes through a disease or disorder, which in turn influences the function of other organs and the challenge is to find the right target for that specific pathway (2). One example is the crosstalk between the pancreas, heart, and kidney organs, leading to vascular and renal diseases as well as diabetic mellitus (DM), which are strongly entwined with each other. Although, many mediators and pathways have been reported in the literature in regard to these diseases, their genesis is still a puzzle (3, 4). In this paper, we reviewed the current incidence, pathways, and the mediators involved in these intricate diseases and made an attempt to compel the common risk factors from the current literature.

## Current Status on Incidence and Prevalence of CKD, DM, and CVD

Since two decades, the shift in the mortality and morbidity is being increased from infectious diseases to non-communicable disease worldwide (5). This rise in the number of patients mostly reflects to vascular and renal disease as well as DM, which are the major public health problem both in developed and developing countries, imposing burden on economy (6).

### Chronic kidney disease

It is defined as a change in the kidney function or structure for more than 3 months that affects the health of an individual irrespective of the cause (7). Based on the glomerular filtration rate (GFR) and albuminuria content it is set into five stages. According to KDIGO guidelines people with GFR ≥90 ml/min/1.73 m^2^ are categorized into first stage where the kidney functions normally, 60–89 ml/min/1.73 m^2^ falls in the second stage with mildly decreased function. The third stage is sub divided into two; people with 45–59 ml/min/1.73 m^2^ and 30–44 ml/min/1.73 m^2^, where the GFR is mildly to severely decreased. In stage 4, the GFR decreases to 15–29 ml/min/1.73 m^2^ and in the fifth stage (<15 ml/min/1.73 m^2^), kidney fails to function. The early stages are asymptomatic and the end-stage is treated by dialysis or transplantation (8). Diabetes, hypertension, cardiovascular diseases (CVD), and cigarette smoking are the common risk factors of chronic kidney disease (CKD) (9, 10). CKD arises due to many pathological insults that affects the renal function and destroys some of the nephrons. As a result, the other nephrons compensate the function of injured nephrons by hyper filtration. Over a period of time, it develops glomerular hypertension, proteinuria, and eventually loss of renal function (11). An increase in the glomerular capillary pressure leads to the destruction of glomerular capillary wall leading to the dysfunction of podocytes that covers the capillaries and allow the permeability of macromolecules (12). These series of insults result in the release of inflammatory mediators and stimulate the proliferation of cells involved in fibrosis. Protenuria impairs the reparative mechanisms resulting in the scar formation due to the accumulation of extracellular matrix (ECM) molecules finally leading to renal failure.

#### Incidence and Prevalence

The incidence of CKD is not clearly known since a large number of patients die due to cardiovascular disease before they progress to end-stage renal disease. In Europe and North America, the prevalence was found to be ~10% and in the US, the prevalence was found to be 13.1% (13). Zhang et al. (14) reviewed 26 population-based studies conducted on the prevalence of CKD and reported that in the elderly, the prevalence was between 23.4 and 35.8%. A meta-analysis by Nitsch et al. studied the association of GFR, albuminuria with mortality, and renal failure by sex, and observed that the increased risk of CKD is equal between both sexes (15).

### Diabetes mellitus

Diabetes mellitus is a group of metabolic disorders resulting from hyperglycemia caused by genetic, molecular, or biochemical factors and activation of renin–angiotensin system (RAS), which eventually leads to the damage of end-organs like kidney (16). Diabetic nephropathy (DN), caused by DM, is one of the progressive kidney diseases characterized by damaged vessels (angiopathy) due to type 1 or type 2 diabetes, hypertension, or dyslipidemia affecting kidney filtering system that might progress to end-stage chronic kidney disease (CKD) (17). DN is also associated with CVD increasing mortality of DM patients (18). The hallmarks of DN are the abnormalities in the glomerulus that alter the structure of podocytes, the decrease in nephrin expression, and the thickening of basement, tubular, and glomerular membranes by the extracellular deposition causing tubulointerstitial and glomerular fibrosis (18).

#### Incidence and Prevalence

According to 2014 statistics, 387 million people are suffering from DM and the prevalence is around 8.3% worldwide, North America being on top (11.4%). 4.9 million deaths were reported in the year 2014 and 46.3% people are undiagnosed (19). DM accounts for 4% of global deaths below the age of 70 years (20). About 25–40% of diabetic patients suffer from DN and/or some degree of CKD (17, 21).

### Cardiovascular disease

Cardiovascular diseases is a group of disorders that is associated with the circulatory system including coronary heart disease (CHD), cerebrovascular disease, peripheral arterial disease, rheumatic heart disease, congenital heart disease, cardiomyopathies, cardiac arrhythmias, and deep vein thrombosis and pulmonary embolism (22). The major risk factors associated with CVD are hypertension, hypercholesterolemia, dyslipidemia, diabetes, smoking, physical inactivity, and obesity (23). Although, few studies have shown that the decrease in the kidney function and higher albuminuria are independent risk factors and not related to diabetes and hypertension (24, 25).

#### Incidence and Prevalence

It is estimated that more than 17 million deaths were reported in the year 2012 globally, which accounts for 31% of all deaths and might increase to 23.3 million by the year 2030. Among these deaths, 7.4 million deaths were due to CHD and the rest were due to stroke, mostly observed in low-income and middle-income countries (22).

## Key Cellular and Molecular Events

### RAAS system

One of the key players that have been implicated in the pathogenesis of cardiorenal disease is renin–angiotensin–aldosterone system (RAAS). In response to the decrease in the renal perfusion pressure, renin (protease) is produced by the juxtaglomerular cells of the kidney. It acts on an inactive peptide called angiotensinogen produced by the liver, converting it to angiotensin-I, which is a rate limiting and initial step of RAAS (26). Apart from juxtaglomerular cells, the highest expression of renin (Prorenin) is observed in the connecting tubules and collecting ducts in diabetes patients (27). Angiotensin-I is catalyzed into octapeptides by angiotensin converting enzyme (ACE-dipeptidyl carboxypeptidase), produced in the lungs and lymphocytes (28), to form angiotensin II, which is an active peptide (29). Angiotensin II is also produced by chymase and cathepsin G, which are independent of ACE activity (30). Furthermore, angiotensin II is cleaved to different peptides by the endopeptidases like ACE2. Recently, many new components have been identified in the RAAS system like angioprotectin, Ang III, IV, V, Ang-(1–7), Angiotensin-A, Alamandine, and their co-factors like vasodilation inducing factor (VIF) (31–34), They are mainly formed by the action of several endopeptidase that act via Mas receptors or Mas-related G-protein-coupled receptor membrane D (MrgD) receptors and they have shown to have vasodilatory effects (33, 35). The effects of Angiotensin II is mediated by the G-protein coupled receptors, i.e., angiotensin type 1 (AT1), angiotensin type 2 (AT2) receptors (30), or the MAS receptor (28). The former being expressed in several tissues like cardiovascular system, kidney, and the sympathetic nervous system (35), and the latter is expressed during fetal life and in adults restricted to the adrenals, ovary, brain, heart, and uterus (36). Angiotensin receptors share overall 30% homology and as a result they have different functions and are adapted to different signal transduction pathways (37). Ang II-AT1 axis has a role in vasoconstriction, cell proliferation, and oxidative stress in the kidney and is counteracted by AT2 receptor, which possesses vasodilatory, antiproliferative, and apoptotic properties (38, 39). Activation of AT1 receptors by angiotensin II in the kidney constricts the efferent arteriole, which results in a decrease of the blood flow that affects the glomerular filtration by raising the glomerular capillary pressure. This in turn results in glomerular injury and an increase in the production of nephrotoxic reactive oxygen species (ROS), profibrotic cytokines, and growth factors. The production of these components stimulates mitogenesis of fibroblast cells that deposit the ECM (renal fibrosis) and inhibits the turnover, leading to CKD (40, 41). Angioprotectin antagonized the contractile actions of Ang II, mediated by the Mas receptor, and Ang A has the same affinity to the AT1 receptor as Ang II, but has higher affinity toward AT2 receptor thus may modulate the harmful effects of Ang II (31, 32). Alamandine acts through the Mrg receptor and shows its vasodilatory effects (33). Furthermore, the recently identified factor vasodilation inducing factor (VIF), a chromogranin peptide, reduces the direct vasoconstrictive effects of Ang II, independent of NO in human plasma (34).

In adrenal cortex, Ang II stimulates the release of aldosterone from zona glomerulosa via AT1, which sequentially regulates the blood pressure, fluid, and electrolyte balance through mineralocorticoid receptors of the distal tubule and collecting duct (42). Aldosterone binds to the mineralocorticoid receptor that regulates the sodium–potassium pumps (43) and since it has cell proliferative and profibrotic properties, it directly increases the expression and production of the profibrotic cytokine “transforming growth factor β” (TGF-β). In kidney disease models, aldosterone synthesis is increased and implicated in the proliferation of fibroblast cells, renal fibrosis, and induction of hypertension due to the sodium overload (44). All these effects synergistically provoke renal damage.

**Figure 1:**
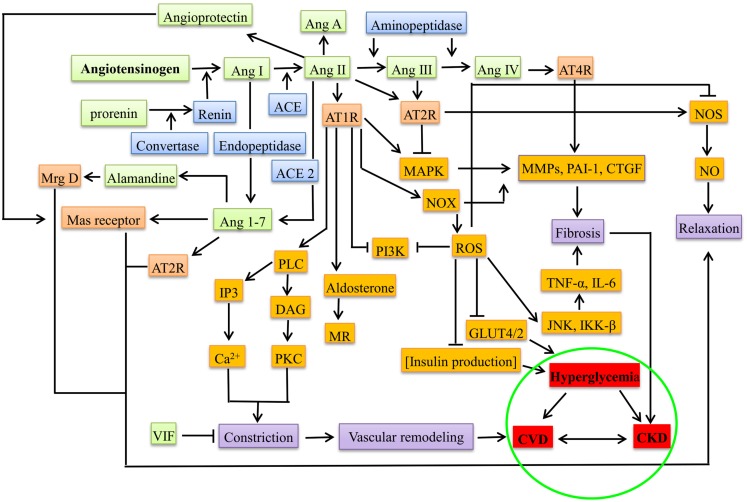
**Overview of RAAS system under pathological conditions (CKD, DM, and CVD)**. The green boxes represent the peptide components of RAAS system, blue represents the enzymes involved, orange represents the receptors, brown represents the downstream molecules/effectors, violet boxes represent the presentation before the disease, and red represents diseased state. Ang, angiotensin; ACE, angiotensin converting enzyme; AT1R, angiotensin type 1 receptor; AT2R, angiotensin type 2 receptor; AT4R, angiotensin type 4 receptor; Mrg D, Mas-related G-protein coupled receptor membrane D; VIF, vasodilation inducing factor; PLC, phospho lipase C; DAG, diacyl glycerol; IP3, inositol triphosphate; PKC, protein kinase C; MR, mineralocorticoid receptor; PI3K, phosphoinositide 3-kinase; MAPK, mitogen-activated protein kinase; NOX, NADPH oxidase; ROS, reactive oxygen species; MMPs, matrix metalloproteinases; PAI-1, plasminogen activator inhibitor-1; CTGF, connective tissue growth factor; TNF-α, tumor necrosis factor alpha; JNK, c-Jun N-terminal kinases; IKK-β, inhibitor of nuclear factor-kappa-B kinase; NOS, nitric oxide synthase; CVD, cardiovascular disease; CKD, chronic kidney disease.

The notion of RAAS acting systemically has been changed by the discovery of local RAAS with paracrine or autocrine action during pathogenesis (32, 45), e.g., during hyperglycemia and proteinuria, each component of RAAS was observed in the proximal tubular cells that synthesize Ang II from angiotensinogen into interstitial and luminal side, leading to the activation of sodium pumps besides aldosterone (46, 47). In β cells of pancreas, the deleterious axis of RAAS, i.e., Ang II-ACE-AT1R-aldosterone increases the oxidative stress, promotes apoptosis, decreases the uptake of glucose (by suppressing the GLUT2 through AT1R), and increases the production of ROS through NADPH oxidase (NOX), thereby decreasing the production of insulin leading to hyperglycemia (4). Figure 1 gives the overview of the mediators in RAAS leading to CKD, DM, and CVD.

Wnt/β-catenin signaling pathway plays an essential role in the organogenesis and tissue homeostasis, which upon activation translocate β-catenin into nucleus, that binds to the T-cell factor (TCF) or lymphoid enhancing factor (LEF) along with other co-factors like CREB-binding protein (CBP), which eventually transcribes the target genes. In healthy subjects, this pathway is silent in kidneys, but in CKD, it is reactivated (48). By bioinformatics approach, Zhou et al. (49) demonstrated that all RAS genes have putative TCF/LEF binding sites at their promoter regions. This is a likely link between the activation of RAS by Wnt/β-catenin during CKD.

### Inflammation

Chronic inflammation is observed in patients with CKD and it contributes to the CVD morbidity and mortality by accelerating the vascular inflammation (50). Release of cytokines at the site of injury recruits the activated immune cells, which further enhances the inflammatory state by producing additional mediators (51). CXCL12 is a chemokine that binds to the CXCR4/7 and has a role in homing of stem and progenitor cells in the bone marrow to the site of injury or into the circulation. CXCL12/CXCR4 axis shows atheroprotective properties by mobilizing the endothelial progenitor cells to the site of injury (52). Local production of Ang II has been implicated in the pathophysiology of inflammation (53). Ang II stimulates the production of proinflammatory molecules like NF-κB by Toll-like receptor 4 in addition to Ang III and Ang IV via AT1 and AT2 receptors (54, 55). Ang II also upregulates the “vascular cellular adhesion molecule-1” (VCAM), “intracellular adhesion molecule-1” (ICAM), and NF-κB, and thereby mediates the production of chemokines like monocyte chemoattractant protein-1 (MCP-1), which recruit the immune cells (56). In mesangial cells, the expression of ETS-1 (E26 transformation-specific sequence), a key regulator of vascular inflammation, is upregulated in the presence of Ang II via ROS production by NADPH oxidase and it participates in inflammation by recruiting T-cells and monocytes/macrophages into the vessel walls (57). In CKD patients, the cytokines; IL-6, IL-1β, and TNF-α are elevated causing the cardiovascular outcomes (58–60). The TAM (Tyro 3, Axl, Mer) ligand-receptor pathway is involved in the regulation of inflammatory processes. This pathway is deregulated in CKD patients and plays a role in atherosclerosis and thrombosis (50). TAM ligands (Gas-6 and Protein S) and receptors are expressed by the innate immune cells that limit the production of proinflammatory cytokines, which are stimulated through toll-like receptors (TLR) by the activation of suppressor of cytokine signaling (SOCS) (61, 62). TAM ligand–receptor signaling is linked to the elevation of cytokines in CKD patients, endotoxemia-mediated TLR activation, chronic monocyte activation, and the involvement of macrophages in atherosclerosis in CKD (50). ROS formation and activation of TLR increases the expression of Gas-6 and shedding of TAM (sTAM) receptors from monocytes binding to Gas-6, which results in the deregulation of TAM receptor signaling, the cytokine, and TLR cascades getting activated in CKD patients and finally chronic inflammation (50). Due to over nutrition and low physical activity, the cells are overloaded with glucose and free fatty acids (FFA), the β cells are unable to produce more insulin, and this causes insulin resistance leading to impaired glucose tolerance (63). The cell avoids citric acid cycle when the caloric intake is more than the required energetics. Acetyl-CoA formed by oxidation of glucose or FFA, combines with oxaloacetate to form citrate, which enters the citric acid cycle, generating an excess amount of NADH that impairs the electron transport in mitochondria resulting in the formation of ROS. The cell avoids it by inhibiting the entry of fatty acids into mitochondria as a result FFA build up in the cytosol (64). Furthermore, the increase in intracellular FFA leads to a decrease in the transportation of GLUT4 to the plasma membrane and therefore the buildup of glucose concentration in the blood (65). Constant exposure to glucose leads to dysfunctioning of β cells and endothelial cells (66). In hyperglycemic conditions, the endothelial cell metabolism is modified and implicated in endothelial dysfunction, e.g., presence of high glucose is activating the NADPH oxidase resulting in the increased production of ROS and an increase in oxidative stress by reducing the entry of glucose-6-phosphate into the pentose phosphate pathway thereby reducing the NADPH formation (67, 68). Excess of glucose activates the arginase uncoupling the endothelial nitric oxide synthase (eNOS) resulting in the increase of superoxide anion production. Increased levels of glucose also diverts to polyol pathway where it is converted to sorbitol by utilizing the NADPH, increasing the ROS production, and activating NF-κB signaling pathway (69–71). Due to changes in the endothelial metabolism, advanced glycation products (AGEs) are formed, which crosslink the extracellular molecules, causing increased vessel stiffness, resulting in vascular complications (72). The AGE products bind to their receptors (RAGE) that are expressed on the monocytes, endothelial cells, and smooth muscle cells. They are involved in the increased expression of scavenger receptor class A in macrophages, which results in the uptake of the oxidized LDL leading to the formation of foam cells, a characteristic feature of atherosclerosis (69). Cholesteryl fatty esters are the main component of foam cells that induce cytotoxicity and are involved in lesion development in atherosclerosis (73). The late stages of macrophages release the lipid content and tissue factors leading to the formation of pro-thrombotic core, resulting in rupture of plaque, leading to blood clot and contributing to myocardial infarction and stroke (73). AGE–RAGE interaction with vascular smooth muscle cells (VSMCs) is involved in cell proliferation calcification process and also activates the NF-κB signaling pathway where it controls the expression of cytokines (71). RAGE also acts as an adhesive receptor in the endothelial cells, attracting the immune cells at the site of injury (74). Nitric oxide (NO) is formed from the arginine by eNOS in endothelium and is involved in vasodilation. Asymmetrical dimethyl arginine (ADMA), an endogenous analog of arginine, which is elevated in atherosclerotic patients competes to bind at the active site of eNOS thereby decreasing the NO (75). ROS produced due to eNOS uncoupling and NADPH oxidase activity triggers the expression of adhesion molecules, transmigration of immune cells, VSMCs proliferation and migration, endothelial apoptosis, and oxidation of lipids (69). Oxidized LDL results in the mitochondrial DNA damage and dysfunction in the endothelial cells, leading to high production of ROS (76). Xu et al. (77) analyzed cohorts of elderly adults and reported that a proinflammatory diet is associated with the systemic inflammation and reduced kidney function.

### Fibrosis

At the site of cell injury or tissue damage, the cells are replaced by the same cell type or with fibrous tissue after the clearance of the inflammatory response. The kidneys have an intrinsic capacity to repair cell death by the de-differentiation and proliferation of tubular epithelial cells. Failure of these processes results in fibrosis during infarction/ischemia or toxic insult (78, 79). Renal fibrosis is a prominent feature of every stage of CKD where an excessive accumulation and deposition of ECM are observed. At the beginning of inflammatory response in interstitium, infiltrates of macrophage population can be observed, which links inversely with the kidney function (80), that could be either deleterious (M1 macrophages) or advantageous (M2 macrophages). This is followed by transdifferentiation of interstitial cell population to myofibroblasts. These myofibroblasts have characteristics of both smooth muscle cells and fibroblast cells, which produce ECM proteins, like collagen or fibronectin, that eventually results in scar formation (81, 82). Myofibroblasts are the primary effector cells involved in both tissue remodeling and fibrosis (83). They are mainly derived from the de-differentiation of resident pericytes and fibroblast cells in the presence of fibrogenic factors that promote cell to cell interaction. These fibrogenic factors are secreted by endothelial cells, epithelial cells, and myeloid leukocytes especially the monocyte-derived cells and they are also dependent on the environmental stimuli like hyperglycemia and hypoxia (84, 85). The M1 and M2 macrophages are derived from monocytes based on the local stimuli. For example, interferon gamma (IFN γ), TLR, tumor necrosis factor, and granulocyte-macrophage colony-stimulating factor stimulate M1 macrophages. M1 macrophages are involved in tissue injury that secretes proinflammatory cytokines such as IL-12, IL-23, IL-1, IL-6, type-I interferon, and also produce reactive oxygen intermediates and nitric oxide. M2 macrophages, on the other hand, play a role in tissue repair and are stimulated by IL-4, IL-10, IL-13, corticosteroids, vitamin D, macrophage colony-stimulating factor, and TGF β (82, 86). Tubular epithelium is involved in the production of ROS and inflammatory mediators that evade the interstitium via basolateral secretion or paracrine pathways (82). They secrete mediators like monocyte chemoattractant protein-1 (MCP-1), IL-8, fractalkine, TGF β, endothelin by triggering megalin receptor-mediated protein endocytosis during protein overload. They also activate specific pathways with other co-receptors, cubilin and amnionless, leading to interstitial inflammation, fibrosis, and loss of nephron (87).

#### TGF-β1

The cytokine TGF-β1 is a prominent and powerful factor mediating myofibroblast activation, which integrates the effects of other fibrogenic factors (88). TGF-β1 is synthesized by all types of cells in the kidney and released in association with the latency-associated peptide (LAP) binding to latent TGF-β-binding protein (LTBP). Upon stress stimuli like hypoxia (89), RAAS (90), oxidative stress (91), and TGF-β1 are activated, binding to the Type-II TGF-β receptor. Type II TGF-β receptor is a kinase that recruits Type-I TGF-β and phosphorylates the downstream molecules like Smad2/3, which in turn forms a complex with Smad4 and then translocate to the nucleus to transcribe the target genes along with other factors (88, 92). TGF-β1 shows profibrotic effects on kidney through different mechanisms where it induces the production of ECM through Smad3 by binding to the promoter region of collagen or Smad-independent pathways. This leads to an inhibition of its degradation by inducing tissue inhibitor of metalloproteinase (TIMPs) and inhibiting matrix metalloproteinases (MMPs). TGF-β1 is also involved in transdifferentiation of different types of kidney cells to myofibroblast cells (88, 93, 94). It has a role in podocytopenia where the podocytes undergo apoptosis and detach from the glomerular basement membrane resulting in the loss of integrity of microvasculature (95). In hyperglycemic conditions, the AGEs and Ang II induce transdifferentiation of epithelial to mesenchymal transition (EMT) mediated through Smad3 phosphorylation (96, 97). TGF-β1 stimulates the differentiation of epithelial, endothelial, and macrophage cells to mesenchymal transition synthesizing ECM (81, 98). Stimulation of myofibroblast with TGF-β1 increases the expression of cannabinoid receptor 1 that increases collagen expression (99). The latent form of TGF-β1 is protective against inflammation and fibrosis by phosphorylating Smad7 (inhibitory Smad) (100). TGF-β1 shows effects via Smad-independent pathways like p38, JNK, extracellular-signal-regulated kinase (ERK), mitogen-activated protein kinase (MAPK), integrin-like kinase (ILK) PI3K/Akt (101–103). TGF-β activates PI3K that phosphorylates Akt and is involved in fibroblast proliferation and ECM deposition (92). Inhibition of PI3K activity results in a decrease in TGF-β-Smad2 phosphorylation and has an effect on EMT and cell migration (104). In mesangial cells, TGF-β1 activates phosphatidylinositol 3-kinase PI3K/Akt signaling, resulting in the mesangial cell hypertrophy and fibrosis in diabetes (105, 106). High glucose levels, protein glycation end-products, Ang II, endothelin-1, and growth factors like PDGF and EGF, induce cell proliferation through PI3K/Akt signal transduction (92).

**Figure 2:**
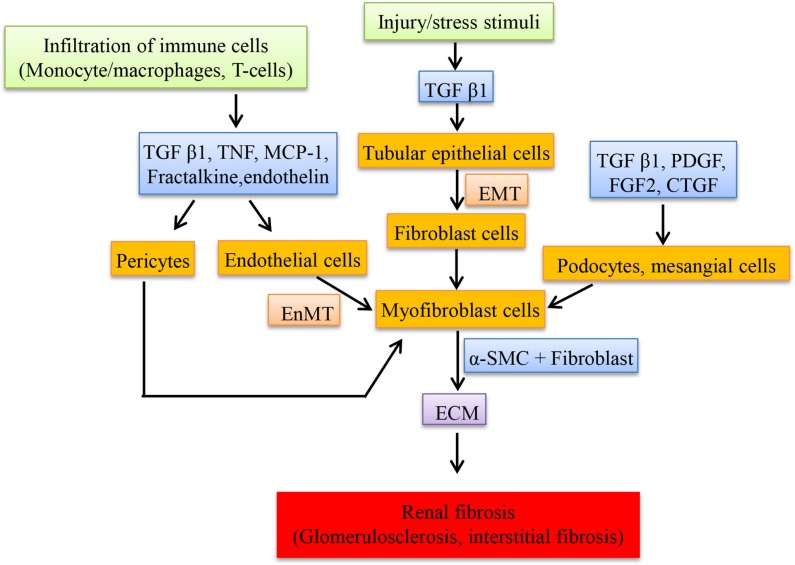
**Schematic representation of cellular mediators involved in renal fibrosis**. The infiltration of macrophages secretes a set of mediators at the injured sites of kidney resulting in the transition of resident cells to myofibroblast cells, which proliferate and secrete the extracellular matrix compounds.

Upregulation of renin and angiotensinogen was observed in glomeruli and involved in the renal scarring or repair by the action of Ang II (107). Transfection of glomeruli with renin and angiotensinogen stimulates the proliferation of fibroblast cells, increases the production of ECM (by inducing the mRNA of proteins like type I procollagen and fibronectin in cultured mesangial cells) and also transcribes collagen type 1 (IV) and 3 (IV) but not type I in cultured proximal tubular cells by Ang II which in turn depends on TGF-β expression (107). Renin alone is able to stimulate the expression of TGF-β in mesangial cells and this could contribute to renal fibrosis despite of Ang II blockade (108). Expression of “connective tissue growth factor” (CTGF), a fibrotic mediator is increased in the kidney induced by TGF-β and Ang II (109). Ang II induces the “plasminogen activator inhibitor-1” (PAI-1) and “tissue inhibitor of matrix metalloproteinases-1” (TIMP-1) via AT1R, which inhibits metalloproteinase affecting the matrix turnover, resulting in accumulation of ECM. PAI-1 is also stimulated by Ang IV via AT4R in proximal tubules thereby playing a role in renal fibrosis (110). In DN, Ang IV is formed by the degradation of Ang II when in high concentrations by different enzymes and this induces PAI-I (111). Most of the fibroblast cells in the interstitium originate from tubular epithelial cells through a process called EMT. This process is mediated by TGF-β and the fibroblast cells are involved in interstitial fibrosis and tubular atrophy (112). Figure 2 shows the cells and the mediators involved in the fibrosis.

All these factors line up, and as a result the kidney loses its function and faces oxidative stress, subsequently leading to the accumulation of toxic substrates. Some of these substrates are involved in the post-translational modification of proteins like carbamylation and oxidation of LDL and HDL, which aggravate the progression of atherosclerosis thus leading to CVD (113, 114). RAAS blockade has been shown to be effective for the treatment of CKD, CVD, DM, hypertension with proteinuria. With an increase in the plasma aldosterone concentrations, the left ventricular mass has shown to be larger in early CKD stages in hypertensive patients (115).

### Uremic toxins

Uremic toxins are in general the waste products that accumulate in the body fluid due to dysfunction of the kidney. They are divided into small water soluble compounds, middle molecules, and protein-bound uremic compounds (116). Among protein-bound uremic toxins, para-cresol sulfate (PCS) and indoxyl sulfate (IS) are linked to the cardiovascular comorbidities (117). p-cresol, a metabolite of p-cresol sulfate showed reduced contraction rates of cardiomyocytes, resulting in the irregular beating mediated by protein kinase C (PKC) by increasing the intracellular calcium levels (118). Furthermore, p-cresol is involved in the increased endothelial micro-particle shedding mediated by Rho-kinase in hemodialysis patients, leading to endothelial dysfunction, which is also observed in acute coronary syndromes, acute ischemic stroke, and venous thromboembolism (119–122).

Indoxyl sulfate also contributes to atherosclerosis and peripheral artery diseases. Indoxy sulfate stimulates NADPH oxidase in endothelial cells, increasing the ROS formation and decreasing the levels of glutathione, thus elevating oxidative stress (123). The uptake of IS through organic anion transporter 3 (OAT 3), further stimulates the expression of “*prorenin receptors*” (PRR) in the aorta of a CKD mouse model, thereby activating aryl hydrocarbon receptors (AhR) and NF-κ B in VSMCs. Activation of PRR by IS promotes cell proliferation and expression of tissue factors like platelet-derived growth factor and receptor (PDGF/R) in VSMCs. It also activates MAPK pathways, thus linking the uremia-induced cardiovascular events (124, 125). Moreover, IS stimulates the production of IL-1, IL-6, and TNF-α in THP-1 cells. Lekawanvijit et al. (126) demonstrated that IS plays a critical role in cardiac remodeling by acting as profibrotic, prohypertrophic, proinflammatory factor, mediated by the activation of MAPK (p38, p42/44) and NF-κ B pathway. IS also upregulates the expression of ICAM and MCP-1 through ROS production, activating NF-κ B pathway in endothelial cells. PCS and IS stabilize the active form of epidermal growth factor (EGFR) and help in dimerization and phosphorylation, leading to an increase in the MMP2/9 expression either directly or by its downstream molecules. Therefore, they are both involved in renal tissue remodeling (127). Under oxidative stress, the protein-bound uremic solutes have an impact on the cardiac tissue architecture as well as on the kidney tissue. For example, uridine adenosine tetraphosphate (Up_4_A), which is synthesized by the endothelial cells acts via purinergic receptors P2Y that activates MEK, ERK1/2 by phosphorylation, and regulates the expression of vascular calcification (VC) mediators like Cbfa, Msx2, Osx, OCN, osteopontin (OPN) leading to the transdifferentiation of vascular smooth vessel cells to osteochondrogenic cells (128, 129). In VSMCs, Up_4_A stimulates the production of ROS by Nox1 dependent way and increases the expression of MCP-1 through phosphorylation of MAPK via P2Y (2) receptor (130). The uremic toxin phenyl acetic acid is associated with arterial vascular properties in patients with CKD that are undergoing hemodialysis (131). It also inhibits the inducible nitric oxide synthase (iNOS) and impairs the macrophage function, thus increasing the incidence of infection in uremia (132, 133).

### Vascular calcification: A link between CKD, DM, and CVD

The mechanism of VC is multifactorial and is poorly understood. The unusual deposition of calcium is observed in media and intima of the vessel, which changes the elasticity and the hemodynamics of the vessel wall that result in stroke and ischemic heart disease (134). This ectopic calcification is noticed in atherosclerosis, diabetes, hypertension, dyslipidemia, and CKD (134). The main presentations of calcification are atherosclerotic lesions, medial calcification, aortic stenosis, and calciphylaxis, which are the most dramatic and often fatal entity. Atherosclerosis, CKD, hyperphosphatemia, and vitamin K-targeting oral anticoagulants have emerged as key contributors to VC.

Vascular calcification is an active and highly regulated process, closely resembling osteogenesis. The factors contributing to the VC are the high levels of calcium and phosphorous due to abnormal bone metabolism, transition of VSMCs to chondrocyte or osteoblast-like cells, imbalance in the levels of inhibitors of calcification, low levels of klotho expression, high levels of parathyroid hormone (PTH), and fibroblast growth factor-23 (FGF-23) expression, Vitamin D, and ECM remodeling (135, 136). VC is characterized by transdifferentiation of VSMCs to osteoblast-like cells where they express a number of bone matrix proteins (137). High plasma calcium phosphate product (Ca × P) contributes to this active process and hyperphosphatemia is considered a key driver of VC in CKD. VC is observed due to imbalance between inhibitory and inducing mediators. The most relevant are OPN, osteoprotegerin (OPG), fibroblast growth factor-23 (FGF-23), bone morphogenetic proteins (BMP), matrix-GLA protein (MGP), CD73, aldosterone, fetuin, pyrophosphate, magnesium, and uremic toxins like Up_4_A. Increased expression of OPN is an indicator of VC and transformation of VSMC to osteoblast-like cells (138). Unphosphorylated OPN enhances whereas phosphorylated OPN decreases VC (138). The amount of OPN can be pharmacologically modified by Mg^2+^ (139, 140). OPG is a decoy receptor for receptor activator of nuclear factor-kappaB ligand (RANKL) that inhibits RANKL-induced VC and bone loss (141). OPG^−/−^ mice display osteoporosis and VC. The phosphaturic hormone FGF-23 activates the FGFR1 receptor in the presence of Klotho (142). Klotho has FGF-23-independent phosphaturic actions. However, it is still unclear whether FGF-23 or Klotho exert direct effects on VSMC phenotype (143). Klotho-deficient mice display accelerated aging, hyperphosphatemia, osteoporosis, and VC. A human klotho mutation was associated with VC in a teenager (144). Both, systemic inflammation and CKD decrease Klotho expression. In CKD, FGF-23 levels increase more than a 100-fold in response to hyperphosphatemia, and elevated FGF-23 levels are predictive of cardiovascular events (145).

Bone morphogenetic proteins are crucial mediators of vascular remodeling and neovascularization (146). BMPs regulate calcification in mesenchymal progenitor cells for cartilage and bone (147) and modify expression of osteoblast markers like alkaline phosphatase in VSMC. The expression of BMPs is increased at VC sites. In addition, BMP and osteoblast homeoprotein Msx2 signaling pathway promotes the differentiation of myofibroblasts into the osteogenic pathway and enhances VC. BMP7 is a calcification inhibitor whereas BMP-2 promotes VC (148). Protein C and S deficit and therapy with vitamin K-targeting anticoagulants are risk factors for calciphylaxis (calcific uremic arteriolopathy) (149). Vitamin K-dependent factors, including proteins C and S, require glutamic acid carboxylation to yield gamma carboxyglutamic residues (Gla) for activation. MGP is a vitamin K2-dependent calcification inhibitor. MGP^−/−^ mice die a few weeks after birth from aortic rupture due to massive calcification. Vitamin K deficiency has been linked to VC in *in vitro* and *in vivo* mouse studies (150). Warfarin, an anticoagulant decreases the active form of MGP and increases VC in CKD rats while vitamin K increased active MGP and prevented VC in warfarin-treated rats (151).

Aldosterone contributes to VC by activating PIT1-dependent osteo-inductive signaling (152). α2-HS-glycoprotein/fetuin levels are decreased in CKD and associate with VC. Uremic toxins, such as IS, induce expression of osteoblast-specific proteins in human VSMC and associate with aortic calcification in CKD, whereas in hypertensive rats, it promotes aortic calcification (153). Leukocyte activation evoked by CKD directly contributes to VC and pyrophosphate acts as an inhibitor of VC (154). A low extracellular pyrophosphate level induces VC in murine model of progeria (155).

Recently, protective effects of VC by magnesium through multiple mechanisms by decreasing the intimal media thickness and aortic pulse wave velocity were described (139). In CKD rats, treatment with a magnesium-based phosphate binder significantly reduced aortic calcification compared to sevelamer-treated rats (156). However, randomized controlled studies should address the impact of magnesium administration in CKD patients.

#### Metabolic Derangements in CKD Modulating VC

In CKD, hyperphosphatemia, iatrogenic hypercalcemia, abnormal levels of PTH and FGF/23, diabetes, and inflammation may contribute to VC.

##### Hyperphosphatemia

High serum phosphate is associated to progression of VC and increased cardiovascular risks in patients with CKD and in dialysis. In the general population, serum phosphorus in the high normal range is associated with increased mortality (157). Exposure of VSMCs to high phosphorus concentrations results in loss of smooth muscle proteins and differentiation into osteogenic cells (158). Osteogenic transdifferentiation may end up in cell apoptosis with production of apoptotic bodies acting as nucleation sites for mineral deposition (159). Hyperphosphatemia-induced nanocrystals upregulate the expression of BMP-2 and OPN VSMC (160). Phosphorus binders are used to reduce phosphorus levels as a key factor in the management of CKD-MBD (mineral and bone disorder). The use of calcium-containing phosphate binders that induce positive calcium balance is associated with increased arterial calcification in the majority of studies (161). The amount of calcium binders required to control phosphate results in positive calcium balance (162) and most studies reveal that the progression of VC is slower with non-calcium-based binders as compared with calcium-containing binders. Excessive load of calcium may favor the development of VC directly or through an inhibition in PTH secretion with the consequent reduction of bone turnover.

##### Hypercalcemia

In CKD patients, hypercalcemia is usually a consequence of excess calcium administration or vitamin D overdosing and is associated to VC. High-calcium medium increases VSMC calcification (159) and potentiates the effect of phosphate. Calcium induces the release of matrix vesicles by VSMC that become calcified in the absence of MGP (163).

##### Hypermagnesemia

In dialysis patients, high serum magnesium concentration was associated with less VC. Low serum magnesium was associated with increased mortality of hemodialysis and non-dialysis CKD patients in observational studies (164). Magnesium-containing phosphate binder-induced positive calcium balance is associated with increased arterial calcification in the majority of studies (165). Many interventional studies showed that magnesium-based compounds are more effective in reducing the phosphate level that improve the survival and progression of VC (166).

##### Vitamin D

Vitamin D may have dose-dependent opposing effects on VC. Vitamin D3 increases calcium transport into the cells and upregulates calcification-enhancing genes, osteocalcin, osterix, and Runx2 (167). Protective effects of Vitamin D3 on VC may be expected by its re-differentiating and anti-inflammatory properties (inhibition of TGFbeta and IL-6) (168), as well as by increasing the expression of VC inhibitors (MGP and Osteopontin).

Experimental studies *in vitro* and *in vivo* have shown that high doses of calcitriol may induce VC (169, 170). Knockout mouse models of FGF-23 and Klotho, which exhibit increased arterial calcification, have elevated calcitriol levels (171). In mice, deficiency in both FGF23 and renal 1-alpha-hydroxylase appear to blunt arterial calcification (171). The effect of calcitriol on VC is mainly due to the increase in phosphate absorption that in renal failure results in hyperphosphatemia.

##### FGF-23-klotho

The hormone fibroblast growth factor-23 (FGF-23) induces phosphaturia and inhibits calcitriol synthesis and PTH production. Serum levels of FGF-23 increase early in the development of CKD. Several clinical studies demonstrated associations between higher levels of FGF23 and VC (172). However, neutralization of FGF-23 in uremic rats results in increased VC (173). A more recent human study including a large population showed that FGF-23 is not associated with VC (174). Some authors have not been able to identify klotho, co-receptor for FGF-23 in human arteries (174). Whether FGF23 has a direct effect on VC is still under debate.

##### Parathyroid hormone

Hyperparathyroidism is a major risk factor for medial calcification in renal disease (175). In uremic animals, PTH infusion produced osteogenic transdifferentiation of VSMCs with increased calcium deposition regardless of phosphate intake. The severity of medial calcium deposition in the aorta correlates with the levels of serum PTH in uremic rats with ovariectomy and with the loss of cortical bone. High and low PTH is associated with high turnover and adynamic bone disease, respectively. They both lead to decreased bone formation, a situations in which the bone is not efficient in incorporating excess calcium and phosphorus (176). This may in turn increase the risk for soft-tissue calcifications.

##### Diabetes mellitus

In diabetic patients, VC is commonly seen in coronary and vascular arteries of lower limbs (177, 178). Monckeberg already described medial artery calcification in an autopsy series that was identified in older age, renal failure, and diabetes as the factors associated with calcifications (179). In patient with diabetes, increased serum creatinine concentration, older age, and poor glucose control are associated with VC and mortality. High cholesterol levels, smoking, and body mass index were not associated with increased VCs. In the Ldlr^−/−^ diabetic mice, a high fat diet produces aortic calcification with concomitant upregulation of osteogenic gene expression (180). Incubation of bovine VSMCs with high glucose led to osteogenic transdifferentiation and calcification (181). In diabetic arteriosclerosis, Msx1 and Msx2 promote vascular mineralization (182). Furthermore, elevation of glucose levels increased the BMP-2/Msx2-Wnt pathway, which promoted osteogenic differentiation (181). Thus, it is likely that high glucose may have direct effects on the osteogenic differentiation of VSMCs and *per se* VC formation.

##### Dyslipidemia

From clinical studies, lipids do not appear to play a major role on VC formation. *In vitro*, HDL inhibits the osteogenic differentiation; among the fatty acids, stearate, promoted mineralization, whereas inhibition of acetyl-CoA carboxylase reduced mineralization (183). As mentioned above, in diabetes, cells are loaded with FFAs. One of the abundantly found long-chain saturated fatty acid is palmitic acid, induces the expression of BMP-2, Msx2, and OPN in human aortic smooth cells through the expression of long chain acyl-coA synthetase 3 and NF-κB that resulted in osteoblastic differentiation (184). Oxidized lipids are involved in VC. Oxidized LDL promotes the calcification process in human coronary artery smooth muscle cells by upregulating the expression of mineral matrix proteins like osterix (Osx) and Runx2 mediated through nuclear factor of activated T cells (185, 186).

##### Inflammation-oxidative stress

Inflammatory cytokines and oxidative stress promote VSMC expression of osteogenic transcription factors. In a murine model of uremia, phenotypic changes in VSMCs occur prior to calcium deposition, accompanied by elastin degradation, possibly due to upregulation of proteases, including matrix metalloproteinases (187).

Oxidative stress and oxidized lipids induce osteochondrogenic differentiation of vascular cells *in vitro*. Oxidative stress induces Cbfa1/Runx2 expression through modulation of PI3-kinase/Akt signaling (188). *In vitro* studies show that TNF-α induces calcification in VSMC. TNF-α promotes phosphate-induced calcification by reducing efflux of a calcification inhibitor, pyrophosphate, and its transport protein, ankylosis protein homolog (189). The important role of oxidant stress on VC is demonstrated in experiments showing that antioxidants block vascular cell calcification *in vitro*.

## Clinical Link of the Diseases

**Figure 3:**
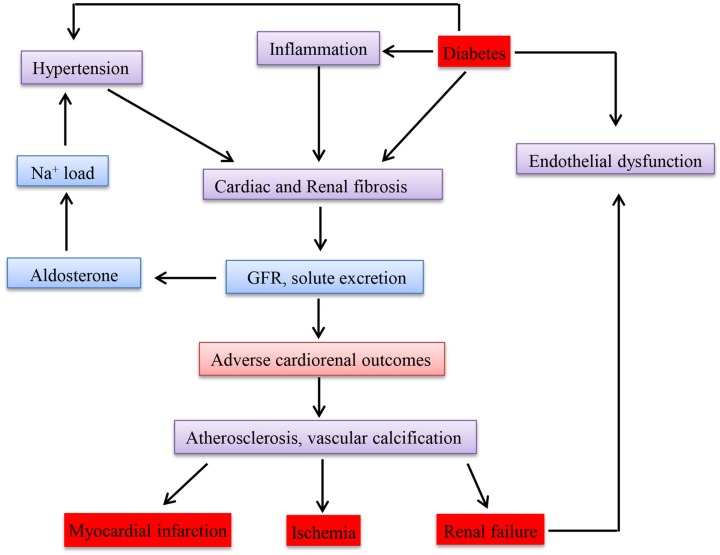
**Schematic representation of clinical link between chronic kidney disease, diabetes mellitus, and cardiovascular disease**.

Epidemiological studies have so far confirmed and reported that there is an association between CKD and CVD (190). Go et al. (191) conducted a study on the relationship between GFR and cardiovascular events in a large group who had not undergone dialysis or transplantation and demonstrated an inverse relationship. The key mechanisms accounting for cardiovascular events apart from the traditional risk factors could be disturbances in mineral metabolism, anemia, ADMA (asymmetric omega NG, NG-dimethylarginine), inflammation, and oxidative stress, which are observed even in CKD. Whether CKD causes CVD or acts as a marker is still controversial (192). Due to disturbances in the metabolism in diabetes, the most effected cells are endothelial and β cells. Since the micro and macro vessels are lined with endothelial cells, the whole vascular system is damaged leading to CKD and CVD. Therefore, disturbance in one of these diseases will affect the other. Figure 3 shows how these diseases are interlinked with each other in a clinical perspective.

## Conclusion

The heart, kidney, and the vascular system are strongly related and help in maintaining hemostasis and cardiorenal equilibrium. Unfavorable outcomes are most common in CKD and for that reason it may deserve a specific approach to evaluate its progression. Due to the advancements in the basic research, many mechanistic pathways have been elucidated in the context of these diseases recently but none of the pathway could interpret the genesis of CKD. Therefore, a key factor in intervention for CKD patients is having an insight on the mechanisms of risk factors associated with it. Our knowledge about the underlying mechanism is increasing, but obviously still at its infancy. However, knowing the key components involved in the pathology of the diseases, their pathophysiological significance and their trend during acute phases, is essential as the basis for new prevention and treatment strategies to combat these diseases in the future.

## Conflict of Interest Statement

The authors declare that the research was conducted in the absence of any commercial or financial relationships that could be construed as a potential conflict of interest.
